# Role of Folate Metabolism in Neurodegenerative Diseases: Insight from Experimental and Clinical Studies

**DOI:** 10.1007/s13668-026-00761-5

**Published:** 2026-04-27

**Authors:** Meenakshi Umar, Karson Franjieh, Amanda Louise White, Elisse Ward-Dones, Saifudeen Ismael, Rose C. Roskey, Jacques Courseault, Gregory Jaye Bix

**Affiliations:** 1 Department of Neurosurgery, Clinical Neuroscience Research Center, Tulane University School of Medicine, New Orleans, LA USA; 2 The Fascia Institute and Treatment Center, 2520 Harvard Ave. Ste 2B, Metairie, LA USA; 3 Tulane Brain Institute, Tulane University, New Orleans, LA USA; 4 Department of Neurology, Tulane University School of Medicine, New Orleans, LA USA

**Keywords:** Folate, one-carbon metabolism, neurodegenerative diseases, Alzheimer's disease, Parkinson’s disease

## Abstract

**Purpose of Review:**

Folate is a key regulator of one-carbon metabolism (OCM), which supports essential physiological processes, including DNA synthesis, repair, methylation, amino acid homeostasis, and redox balance. It is also crucial for brain health throughout life, from neural tube formation during early development to neurotransmitter synthesis, myelination, neuronal development, synaptic plasticity and cognitive function during later stages of life. Disruption of folate-mediated OCM (FOCM) can adversely affect brain health and contribute to neurodegeneration. In this review, we summarize current evidence linking FOCM dysregulation to neurodegenerative diseases, emphasizing disease-specific mechanisms and the therapeutic potential of modulating folate metabolism, as evidenced by experimental and clinical studies.

**Recent Findings:**

Disruption of FOCM can lead to oxidative stress, impaired methylation, excitotoxicity, and neuroinflammation, thereby contributing to neurodegenerative diseases. In Alzheimer’s disease, impaired FOCM promotes amyloid-β accumulation, tau pathology, cognitive decline, and vascular dysfunction, consistent with low folate and elevated homocysteine observed clinically, though supplementation outcomes remain mixed. In Parkinson’s disease, folate deficiency and hyperhomocysteinemia exacerbate motor deficits and dopaminergic neurodegeneration via oxidative stress, mitochondrial dysfunction, and NLRP3-mediated inflammation and combined folate and vitamin B12 supplementation may reduce levodopa-associated risks. Elevated homocysteine and aberrant FOCM have also been reported in Amyotrophic Lateral Sclerosis, Multiple Sclerosis, and Huntington’s disease and are associated with neuroinflammation, demyelination, neuronal loss, and severe disease phenotypes in these conditions.

**Summary:**

Overall, maintaining optimal folate levels may be a promising strategy to support brain health and reduce the risk of neurodegenerative disorders.

## Introduction

Folate, also known as pteroylglutamic acid or vitamin B9, plays a key role in one-carbon metabolism (OCM), which is essential for several physiological processes including DNA synthesis, repair, methylation, amino acid homeostasis and redox defense [1]. Beyond this fundamental role, folate is essential for normal brain function, influencing neurotransmitter synthesis, myelination, neuronal development, synaptic plasticity and cognitive function [2, 3]. Folate deficiencies can lead to neuronal damage [4] and cognitive decline [5] and are associated with increased risk of neurodegenerative diseases such as Alzheimer’s disease (AD) and Parkinson’s disease (PD) in epidemiological studies [6–9]. Despite its recognized importance in brain health, the precise mechanisms by which folate deficiency contribute to neurodegeneration remain incompletely understood. In this review, we provide a comprehensive overview of the diverse roles of folate in the brain and examine emerging evidence for its involvement in the pathogenesis of neurodegenerative diseases such as AD, PD, Amyotrophic Lateral Sclerosis (ALS), Multiple Sclerosis (MS), and Huntington’s disease (HD).

## Folate and its Dietary Sources

Folate is a water-soluble vitamin involved in the synthesis of amino acids and nucleotides, and is essential for the body, particularly during anabolic periods of growth and development. Folate requirements are particularly high during pregnancy, lactation, and fetal development [10]. The insufficient folate intake during pregnancy is linked to congenital malformations and developmental delays [11, 12]. Structurally, folate is composed of a pteridine ring, para-aminobenzoic acid, and a variable number of glutamic acid residues. Humans cannot synthesize folate and obtain it from natural sources of food, food fortified with folic acid (the synthetic fully oxidized monoglutamate form of folate), and through dietary supplements such as 5-methyltetrahydrofolate (5-MTHF) or 5-formyl tetrahydrofolic acid (folinic acid or leucovorin, an active form of folate). Folate occurs naturally in dark green leafy vegetables, beans, and legumes, primarily in the polyglutamate form. Before absorption, it is hydrolyzed to the monoglutamate form by folate hydrolase located on the small intestinal enterocytes [13]. Monoglutamate folate is absorbed through the reduced folate carrier (RFC), folate receptors (FR), and the proton-coupled folate transporter (PCFT) and is converted into its active forms, dihydrofolate (DHF) and tetrahydrofolate (THF), through OCM within the cell.

## Folate-mediated One-carbon Metabolism (FOCM)

FOCM comprises a network of biochemical reactions that generate and transfer one-carbon units for various biosynthetic processes [1]. These reactions occur in different cellular compartments such as mitochondria, cytosol and nucleus [14]. In mitochondria, serine oxidation produces formate, which is transported to the cytosol. In the cytosol, formate is converted to 10-formyl-THF and subsequently to 5,10-methylene-THF, completing a redox cycle. The exchange of folate intermediates between compartments maintains key anabolic pathways, such as the synthesis of purines and thymidylate and the methionine cycle [1].

**Fig. 1:**
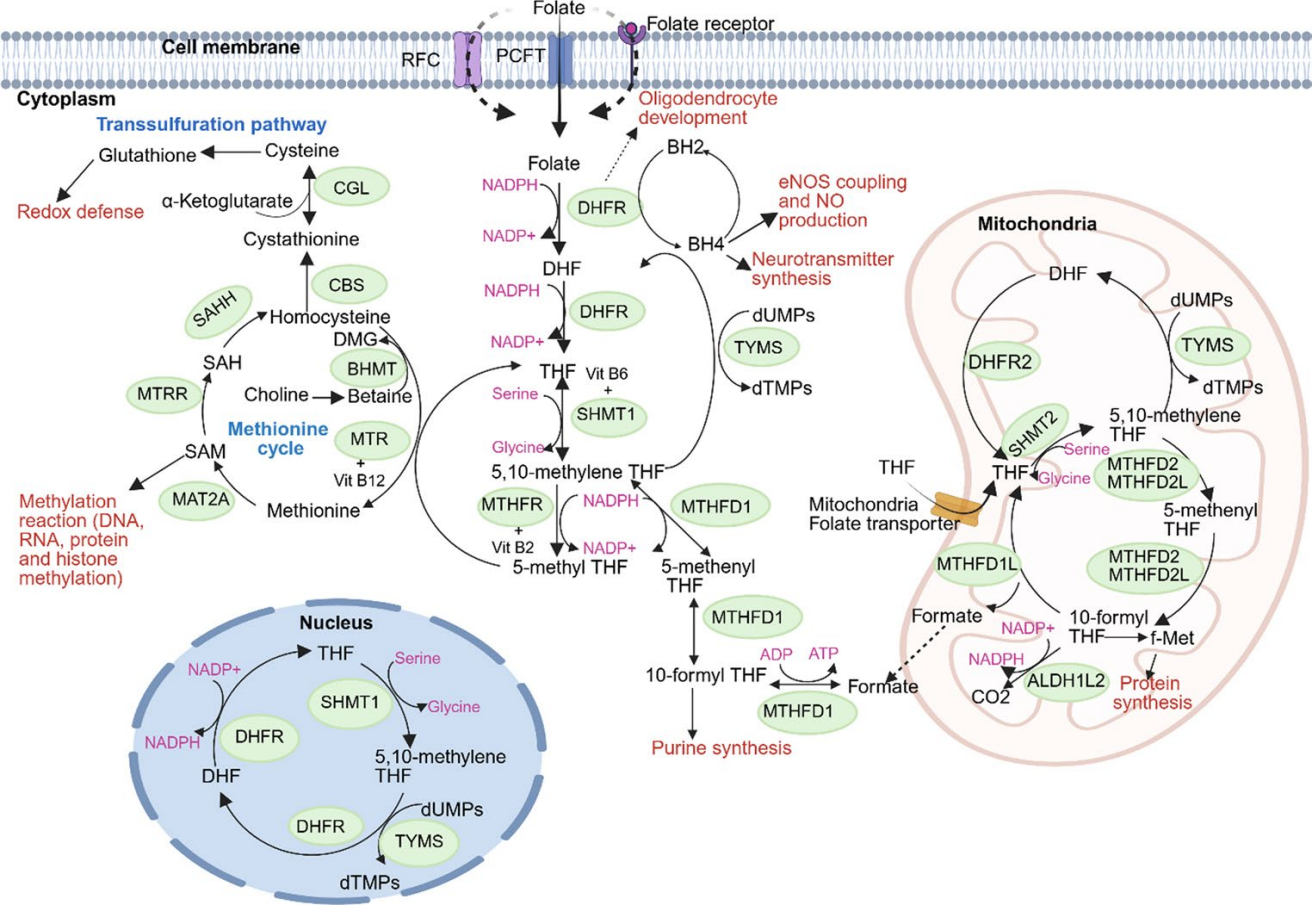
Overview of folate-mediated one-carbon metabolism (FOCM). Compartmentalization of FOCM in mitochondria, cytosol, and nucleus. Following cellular uptake, dietary folate is converted to DHF and then to THF by DHFR, and subsequently to 5,10-methylene-THF by SHMT. Formate from mitochondria can also generate 5,10-methylene-THF via MTHFD1. Cytosolic 10-formyl-THF supports purine synthesis, while 5,10-methylene-THF supports TYMS-mediated dTMP synthesis. Increased DHFR expression supports BH4 regeneration for neurotransmitter synthesis and eNOS coupling. 5,10-methylene-THF is reduced to 5-MTHF by MTHFR to support the methionine cycle, producing SAM for cellular methylation. Homocysteine is either recycled or enters the transsulfuration pathway via CBS, generating cysteine and glutathione. The figure was created using BioRender (biorender.com)

In cytosol, the folate cycle begins with dietary folate being converted into DHF, which is subsequently reduced to THF by the enzyme dihydrofolate reductase (DHFR). THF is then converted into 5,10-methyleneTHF via serine hydroxymethyltransferase (SHMT1 or SHMT2α), a reaction that simultaneously converts serine to glycine and requires vitamin B6 as a cofactor. Alternatively, in the cytosol, 5,10-methyleneTHF can be derived from formate transported from mitochondria which can be converted to 10-formyl-THF and subsequently to 5,10-methyleneTHF by methylenetetrahydrofolate dehydrogenase 1 (MTHFD1). Cytosolic 10-formyl-THF is essential for purine biosynthesis. Whereas 5,10-methyleneTHF is used as a methyl donor by Thymidylate synthase (TYMS) to convert deoxyuridine monophosphate (dUMP) into deoxythymidine monophosphate (dTMP), generating DHF to re-enter the cycle. Additionally, 5,10-methyleneTHF can be reduced to 5-methyltetrahydrofolate (5-MTHF) by methylenetetrahydrofolate reductase (MTHFR), using vitamin B2 as a cofactor. 5-MTHF can enter the methionine cycle by donating a methyl group to homocysteine (Hcy) to regenerate methionine via methionine synthase (MTR), which requires vitamin B12 in the form of methylcobalamin. Methionine is converted to S-adenosylmethionine (SAM) by methionine adenosyltransferase 2 A (MAT2A), which serves as the universal methyl donor for DNA, RNA, protein, and histone methylation reactions. SAM is demethylated to S-adenosyhomocysteine (SAH), which is hydrolyzed by S-adenosylhomocysteine hydrolase to regenerate Hcy. In the transsulfuration pathway, Hcy is catalyzed by cystathionine beta-synthase (CBS) in the presence of vitamin B6 to produce cysteine and, consequently, antioxidant glutathione (Fig. 1).

In mitochondria, THF is imported via a mitochondrial folate transporter and converted into 5,10-methylene-THF by serine hydroxymethyltransferase 2 (SHMT2), which oxidizes serine to glycine while transferring a one-carbon unit to THF [15]. This mitochondrial pool of 5,10-methylene-THF is utilized both for deoxythymidine monophosphate (dTMP) synthesis and for conversion into 10-formyl-THF via methylene-THF dehydrogenase 2 (MTHFD2) or its isoform MTHFD2L. Mitochondrial 10-formyl-THF contributes to mitochondrial protein synthesis by providing formyl groups for formyl-methionine tRNA. It can also be oxidized to CO₂ through the action of mitochondrial 10-formyl-THF dehydrogenase (ALDH1L2). Alternatively, formate can be generated from 10-formyl-THF by methylenetetrahydrofolate dehydrogenase 1-like (MTHFD1L) (Fig. 1).

Within the nucleus, folate cycle involves the conversion of THF into key folate derivatives, including 5,10-methylene-THF and 5-methyl-THF. To support this cycle, several folate-related enzymes such as TYMS, DHFR, MTHFD1, SHMT1, and the nuclear isoform SHMT2, are transported from the cytoplasm into the nucleus. This translocation occurs at the onset of S-phase to enable de novo dTMP synthesis required for DNA synthesis [16] (Fig. 1).

In summary, mitochondrial OCM primarily uses serine as a one-carbon source and plays a critical role in supporting biosynthetic processes such as nucleotide synthesis, protein synthesis and methylation, particularly under conditions of high proliferative demand and cellular stress [17]. The cytosolic pathway depends on one-carbon units such as formate (exported from mitochondria), amino acids (serine and glycine), and dietary folate to support DNA synthesis, methionine cycle, cellular redox balance and methylation [18]. In addition, the nuclear folate cycle is required for DNA replication, repair, and epigenetic regulation within the nucleus [16].

## Role of Folate in Brain Health

Folate is crucial for brain health throughout life [19, 20]. It is indispensable for cell proliferation and tissue growth during fetal development, and folate deficiency during the periconceptional period is linked to a higher risk of neural tube defects (NTDs) in the developing fetus [21]. Studies showed that NTDs defects, such as spina bifida, meningomyelocele, encephalocele, and anencephaly, stem from defects in neural cell proliferation, differentiation, and death [22, 23]. In addition, deficiencies in maternal OCM resulting from either MTHFR deficiency or reduced intake of folate or choline, in pregnant mice can lead to impaired short-term memory and increased apoptosis in the hippocampus of offspring [24]. Consistent to these findings, studies have shown that folic acid supplementation during pregnancy significantly reduces risk of NTDs and increases cognitive performance post-birth in humans [25–27]. In addition, genetic defects in folate metabolism and its transport can cause megaloblastic anemia and a variety of neurological disorders, including developmental delays, intellectual disability, seizures, and ataxia, underscoring the vital role of folate in brain development and function during both fetal and postnatal stages [28, 29]. For instance, hereditary folate malabsorption, caused by mutations in the Proton-coupled folate transporter (PCFT )gene (SLC46A1), impairs folate transport in the intestine and brain, leading to systemic folate deficiency with symptoms such as megaloblastic anemia and neurological impairments, including developmental delays and seizures [30]. The critical role of folate in the brain is further recognized by the cerebral folate deficiency, which is characterized by low cerebrospinal fluid (CSF) folate levels in the absence of systemic folate deficiency. Cerebral folate deficiency can cause progressive neurologic deterioration, such as psychomotor retardation with hypotonia and ataxia, dyskinesias, cognitive impairment, and seizures [31]. It can occur as a result of folate receptor-alpha (FRα) autoantibodies or due to loss-of-function mutation in the FOLR1 gene, leading to impaired folate transport across the choroid plexus to the brain [32]. Recently, a meta-analysis has linked FRα autoantibodies with autism spectrum disorders (ASD) and has reported improvement in ASD symptoms with leucovorin supplementation [33], with a more pronounced beneficial effect in ASD children with FRα autoantibodies [34, 35]. In addition, multiple studies have reported that maternal intake of folic acid and multivitamin supplements before and during pregnancy is associated with reduced risk of ASD in offspring [36, 37]. In contrast, a few reports suggest that excessive multivitamin supplementation during pregnancy and extremely high maternal plasma levels of folate at the time of birth increase the risk of ASD, suggesting a potential “U” shaped relationship between maternal multivitamin use and ASD risk in offspring [38]. However, this study measured only total plasma folate 2–3 days post-delivery and did not assess unmetabolized folic acid, limiting conclusions about whether the observed risk is specific to unmetabolized folic acid or to overall folate levels.

In addition to its critical role in early development, folate is essential for maintaining neurological health throughout later life and for healthy brain aging [20]. Folate can modulate neurotrophic factors that support brain development and function. Sufficient folate levels are required for the synthesis and function of neurotrophins, including brain-derived neurotrophic factor (BDNF) and nerve growth factor (NGF) [39, 40]. As neurotrophins are vital for neuron survival, synaptic plasticity, and the establishment of neural networks [41–43], reduced BDNF and NGF levels caused by folate deficiency can potentially contribute to cognitive deficits and an elevated risk of neurodevelopmental and psychiatric conditions [44, 45]. Folic acid may also play a role in the repair of peripheral nerve injury by promoting the proliferation and migration of Schwann cells and the secretion of NGF [46].

Folate is essential for biosynthesis of monoamine neurotransmitters such as dopamine, serotonin and norepinephrine [47]. These neurotransmitters are essential for cognitive function and basic physiological processes like sleep, appetite, thermoregulation and pain modulation [48, 49]. Therefore, disruption in folate metabolism can affect mood regulation, reward processing, and emotional stability by reducing neurotransmitter synthesis. SAM derived from methionine, functions as the main source of methyl group in the central nervous system. These methyl groups regulate the enzymes and factors essential for producing tetrahydrobiopterin (BH4), a key cofactor required for synthesizing monoamine neurotransmitters from amino acids. When folate metabolism is impaired, SAM production declines, leading to lower levels of neurotransmitters in the CSF [50].

Folate plays a crucial role in oligodendrocyte maturation and myelination [51]. Oligodendrocytes myelinate axons throughout the central nervous system, improving signal speed and transduction. Weng et al. have shown that folate promotes phosphorylation of adenosine monophosphate-activated protein kinase (AMPK) via increased DHFR expression, which in turn supports growth and development of oligodendrocytes [51]. Methotrexate, an inhibitor of DHFR, causes oligodendrocyte death and differentiation defects, further confirming the role of folate in oligodendrocyte development [51, 52]. In addition, folate regulates the synthesis of phosphatidylcholine and sphingomyelin, two major phospholipids of myelin, via SAM [53].

Folic acid plays an important role in maintaining brain endothelial cell function by increasing endothelial nitric oxide synthase (eNOS) coupling and function [54]. Folic acid can upregulate DHFR expression and activity, which in turn enhances the regeneration of BH4 from its oxidized form, dihydrobiopterin (BH2) [55, 56]. eNOS uses BH4 as a cofactor to remain in its “coupled” state and produce nitric oxide (NO), which plays a key role in maintaining vascular tone, cerebral blood flow, and neuronal signaling. Therefore, low folate levels can result in BH4 deficiency, leading to uncoupling of eNOS and generation of superoxide instead of NO, contributing to endothelial dysfunction and neurovascular damage [57]. In addition, folic acid is reported to affect phosphorylation of eNOS [57]. Thus, folic acid improves eNOS coupling and function, supporting NO production and protecting brain endothelial function. These studies linking the folic acid-DHFR-BH4-eNOS pathways were conducted in experimental models. In humans, randomized controlled trials and meta-analyses have similarly shown that folic acid supplementation can improve endothelial function [58, 59]. However, DHFR activity in human tissues is relatively low and shows considerable interindividual variability, suggesting that the benefits of folic acid supplementation may be limited by potential saturation of DHFR [60]. Overall, folate plays a pleiotropic role in brain health, and dysregulation in folate metabolism can have a detrimental effect on the brain. For instance, Mthfr deficiency which disrupts folate metabolism is associated with structural, cognitive, and behavioral impairments in both humans and mice [61–64]. In addition, mild hyperhomocysteinemia (HHcy) associated with methionine synthase reductase (Mtrr) deficiency results in global DNA hypomethylation, disturbances in choline metabolism and short-term memory impairments in mice [65]. Moreover, increasing evidence suggests the role of impaired folate metabolism in neurodegenerative processes [20, 66], underscoring its importance in maintaining neurological function.

## Mechanism of Folate Deficiency-mediated Neurodegeneration

The precise mechanisms by which folate deficiency contribute to neurodegeneration remain unclear, though it is largely attributed to disruptions in OCM. This disruption triggers a series of damaging effects in the brain through HHcy, oxidative stress, impaired DNA repair, diminished methylation, and neuroinflammation [20, 66]. Among these, HHcy is one of the most extensively studied mechanisms underlying folate-related neurodegeneration. HHcy levels can cause neuronal death by inducing DNA damage, which activates poly-ADP-ribose polymerase (PARP) and depletes nicotinamide adenine dinucleotide (NAD⁺) and adenosine triphosphate (ATP) ultimately resulting in loss of mitochondrial membrane potential and caspase activation [67]. HHcy can also contribute to neuronal toxicity through excitotoxic mechanisms [68] primarily by overstimulating N-methyl-D-aspartate (NMDA)-type glutamate receptors. The hyperstimulated NMDA receptor causes excessive Ca²⁺ influx and subsequent rapid, sustained phosphorylation of extracellular signal-regulated kinase (ERK) mitogen-activated protein kinase (MAPK) [69]. Activation of ERK MAPK can trigger a biphasic response of p38 MAPK, consisting of an initial rapid activation followed by a delayed, sustained activation, during homocysteine (Hcy)-induced excitotoxicity. In fact, inhibition of ERK phosphorylation can reduce Hcy-mediated neuronal cell death [69]. This suggests that ERK and p38 MAPK signaling pathways play a critical role in Hcy-induced NMDA receptor stimulation, ultimately contributing to neuronal death [70]. It is important to note that the effect of Hcy on NMDA receptors depends on glycine concentration. Hcy can both inhibit and activate NMDA receptors, depending on glycine concentration. At normal glycine levels, low Hcy acts as a partial antagonist and may be neuroprotective [68, 71]. However, when Hcy levels are elevated, or when glycine levels rise (e.g., after stroke or trauma), even physiologically low Hcy levels become excitotoxic by activating NMDA receptors [72]. Hcy-mediated excitotoxicity can also result from mitochondrial dysfunction and oxidative stress, which are preceded by PARP activation and NAD depletion [67].

Another mechanism by which folate deficiency and HHcy contribute to neurodegeneration is through disruption of the glutathione-mediated redox defense. Folate deficiency disrupts the transsulfuration pathway by reducing cysteine availability, a key precursor for glutathione synthesis, through impaired methionine cycle function, or by decreasing the activity of CBS, which converts Hcy into cysteine [73]. Elevated Hcy levels and folate deficiencies can impair antioxidant glutathione peroxidase (GPx) activity [74]. GPx uses glutathione as a cofactor and protects cells from oxidative damage by reducing harmful peroxides. However, the reports on the effect of folate deficiency on the antioxidant glutathione levels are inconsistent. In neuronal cultures, folate deficiency decreases levels of reduced glutathione, leading to elevated oxidative stress, increased reactive oxygen species (ROS) production, and elevated amyloid-β–induced apoptosis [75]. In contrast, folate deficiency in Apolipoprotein E (ApoE)-deficient mice is reported to increase glutathione levels, possibly as a compensatory response to oxidative stress in the brain [76]. A recent study by Wang et al. showed that folic acid supplementation mitigates age-associated neurodegeneration by enhancing extracellular cystine uptake and intracellular glutathione synthesis leading to activation of solute carrier family 7 member 11-glutathione-GPX_4_ antioxidant pathway [77].

As mentioned in previous sections, folate is required for the synthesis of SAM, the primary methyl donor in cellular methylation reactions. Folate deficiency leads to reduced SAM and accumulation of SAH, resulting in global hypomethylation. This hypomethylation can alter expression of genes involved in neuroprotection, processing of amyloid precursor protein (APP), tau phosphorylation pathways, and inflammatory mediators [78]. These epigenetic disruptions can contribute to the pathogenesis of AD, PD, and other neurodegenerative disorders by promoting neurodegeneration and cognitive decline [78, 79].

Neuroinflammation can be an important mechanism linking folate deficiency to neurodegeneration. Low folate status and HHcy can activate microglia and astrocytes, leading to the release of pro-inflammatory cytokines (e.g., tumor necrosis factor-alpha (TNF-α) and interleukin 6 (IL-6) [80]. Folate deficiency can also activate nuclear factor kappa-light-chain-enhancer of activated B cells (NF-κB) pathway, which leads to elevated levels of IL-6 and C-reactive protein in peripheral blood, as well as increased NF-κB expression in the brain of zebrafish [81]. This neuroinflammatory response is associated with cognitive impairment, upregulation of tau and reduced synaptopodin in zebrafish [81]. Thus, neuroinflammation associated with folate deficiency can lead to oxidative stress, mitochondrial dysfunction, and excitotoxicity, all of which accelerate neuronal injury.

Based on these mechanistic understandings, it is evident that folate deficiency can play an important role in the development of major neurodegenerative diseases such as AD, PD, MS, ALS, and HD. These conditions share common pathologies, including progressive loss of neurons, oxidative stress, impaired DNA repair, and abnormal protein aggregation. These processes can be exacerbated by folate deficiency and OCM disruption. Clinical and epidemiological research also suggest associations of low folate levels and elevated Hcy with increased risk of AD and PD [6–8]. In addition, folate deficiency has been linked to dopaminergic system dysfunction and increased oxidative stress in PD [82]. Thus, folate deficiency may contribute to both susceptibility and disease progression in these neurodegenerative disorders. In the next section, we will explore the evidence connecting FOCM with AD, PD and other neurodegenerative diseases.

## FOCM and Alzheimer’s Disease

AD is a progressive neurodegenerative disorder that primarily affects individuals over the age of 65. It is the most common cause of dementia and is marked by cognitive decline, memory impairment, and behavioral changes [83]. AD is characterized by chronic inflammation, neuronal death, synapse loss, and the accumulation of amyloid-beta (Aβ) plaques and tau neurofibrillary tangles. The Aβ plaques arise extracellularly following aberrant cleavage of amyloid precursor protein (APP) by beta- and gamma-secretases, whereas tau, a microtubule-associated protein, becomes abnormally hyperphosphorylated, detaches from microtubules, and aggregates into intracellular tangles [84]. Impaired clearance of Aβ further promotes its accumulation. Accumulation of plaques and neurofibrillary tangles disrupts neuronal and synaptic function, leading to cognitive decline and neurodegeneration. Genetic predisposition, environmental exposures, and lifestyle factors are key contributors to the development and progression of AD. Deficiencies in nutrients such as various vitamins, including folate, are also recognized as risk factors for AD [85, 86].

### Clinical and Epidemiological Research Linking FOCM and AD

Several clinical studies have linked folate/folic acid deficiency and HHcy to an elevated risk of AD in cross-sectional, case-control studies [6, 8, 9] and longitudinal cohort studies [87, 88]. For example, a longitudinal study of 814 Italian subjects reported that elevated plasma Hcy levels and reduced serum folate concentrations are independent predictors of dementia and AD risk [87]. Similarly, the Framingham study, which followed 1,092 dementia-free individuals, identified HHcy as an independent risk factor for dementia and AD [88] underscoring the importance of FOCM in AD pathogenesis.

Genetic studies have also examined the role of polymorphisms in folate pathway genes with susceptibility to AD. Among these, the *MTHFR* C677T variant has been most extensively studied, although findings from individual studies remain inconclusive, possibly due to underpowered cohorts [89, 90]. However, two separate meta-analyses of these studies showed that C677T increases susceptibility to late-onset AD, particularly in Asian populations and among APOE ε4 carriers [91, 92], while others confirmed its association with AD overall [93] or within the Asian cohort [94]. Functionally, the C677T variant reduces MTHFR enzyme activity by approximately 34% in CT heterozygotes and by up to 70–75% in TT homozygotes compared with CC wild types [95]. Another common variant, *MTHFR* A1298C, has also been studied in relation to AD risk, although the results remain inconsistent [96, 97].

In addition to *MTHFR*, limited studies suggest associations between other folate cycle gene variants and AD. For example, variants in *MTHFD1* (rs1076991 and rs2236225) have been reported as strong susceptibility markers for AD [98], although another study found only a weak association between the *MTHFD1* rs2236225 polymorphism and AD [99]. A large genome-wide association study (GWAS) further showed the link between mitochondrial FOCM and AD by identifying a novel association between *MTHFD1L* rs11754661 and late-onset AD, independent of the APOE risk variant rs2075650 [100]. This association was later confirmed in an independent Chinese cohort [101].

Folate deficiency and *MTHFR* C677T polymorphism also appear to affect brain structure and perfusion in AD. In a study of 102 early symptomatic AD (with or without mild cognitive impairment) and 71 cognitively unimpaired individuals, *MTHFR* 677T allele was associated with hypoperfusion in the left precuneus of AD patients compared to non-carriers [102]. Additionally, a synergistic effect of low folate concentration and the *MTHFR* 6777T allele was linked to the atrophy of specific hippocampal subregions in AD patients [102]. Disruption in folate and Hcy metabolism may also contribute to amyloid pathology in AD brain. A study reported an association of elevated CSF Aβ1–42 levels with high plasma Hcy and low vitamin B12, as well as with high CSF Hcy and SAH along with low 5-methyltetrahydrofolate (5-MTHF) [103]. Overall, these findings suggest the critical role of low folate, elevated Hcy and variants in *MTHFR* (C667T) and *MTHFD1L* (s11754661) as risk factors of AD, which can accelerate cognitive decline and neurodegeneration in AD through both metabolic and structural mechanisms.

Several clinical trials have also investigated the effects of FOCM metabolites and cofactors in AD, but the results remain inconsistent. A multi-center, double-blind, controlled clinical trial (NCT00056225) evaluated the effects of high-dose folate, vitamin B6, and vitamin B12 (5 mg folate, 25 mg vitamin B6, and 1 mg vitamin B12) in 409 participants with mild to moderate AD, who had normal baseline levels of these vitamins and Hcy [104]. While the treatment successfully lowered Hcy levels, it did not improve primary cognitive outcomes over 18 months. Instead, an increased incidence of adverse events, including depression, was observed among participants receiving supplements [104]. Other clinical trials also did not find a beneficial effect of folic acid supplementation in combination with methylcobalamin or with acetylsalicylic acid and vitamin B6 (pyridoxine) in cognition of AD or vascular dementia patients with normal Hcy or mild HHcy [105, 106]. In addition, a double-blind placebo-controlled study (NCT00597376) involving 100 subjects with memory complaints but normal Hcy levels, participants were treated with Cerefoli NAC (containing L-methylfolate, methylcobalamin, and N-acetylcysteine) in combination with a standardized multivitamin for six months. The study reported no beneficial effect of Cerefolin NAC on glutathione levels, inflammatory cytokines or Aβ42/Aβ40 ratio compared to a placebo (https://www.clinicaltrials.gov/study/NCT00597376?tab=results). However, Cerefolin was observed to delay the progression of hippocampal and cortical atrophy in AD and related disorders patients as well as forebrain parenchymal atrophy in cerebrovascular disease patients with HHcy [107]. Similarly, two clinical trials conducted in China have shown that folic acid, either alone [108] or combined with vitamin B12 [109], improved cognitive function, increased SAM/SAH levels, and reduced TNFα in AD patients. In a separate small phase II double-blind clinical trial (NCT01320527) involving 106 AD patients, a nutraceutical formulation containing folate, alpha-tocopherol, vitamin B12, S-adenosyl methionine, N-acetyl cysteine, and acetyl-L-carnitine improved performance on the Dementia Rating Scale, with a non-significant trend toward improvement on the Neuropsychiatric Inventory. The formulation appeared more effective in individuals at earlier stages of AD than in those who began treatment later [110]. This nutraceutical formulation maintained the cognitive performance of 24 AD patients in a one-year open-label study [111] as opposed to the routine cognitive decline of participants receiving a placebo [112]. Taken together, these findings suggest that vitamin supplementation, including folate/folic acid, may be more beneficial for AD patients with elevated Hcy levels or coexisting cerebrovascular disease. Conversely, supplementation in AD patients with normal Hcy should be approached with caution due to potential side effects.

### Experimental studies

Both animal and in-vitro studies provide additional evidence for relationship between AD and FOCM. In amyloid precursor protein (APP) mutant transgenic mice, folic acid deficiency leads to enhanced impaired DNA repair and hippocampal neurodegeneration [113]. Similarly, hippocampal cultures maintained in folic acid-deficient or high-Hcy condition exhibited increased cell death and susceptibility to Aβ-induced toxicity [113]. In cortical neurons and SH-SY5Y cells, folate deficiency through Hcy accumulation induces AD-like pathology such as increased cytosolic calcium (via NMDA receptor activation), ROS, phosphorylated tau and apoptosis, suggesting folate/folic acid deficiency exacerbate AD neuropathology by elevating oxidative stress and excitotoxicity [75]. Consistent with these findings, Crivello et al. showed that folate deficiency in APP/presenilin (PS1) mice accelerates AD related pathologies such as worsened memory deficits, increased Aβ plaque burden and reduced sphingomyelin in cortex [114]. In addition, increased levels of APP, PS1, and Aβ proteins were observed in the hippocampus of folate-deficient APP/PS1 AD mice in another study, which were mitigated by folic acid supplementation [115].

Defects in FOCM enzymes further contribute to AD pathology by increasing vascular and neuronal vulnerability. For example, Bahous et al. showed that *Mthfr*^*+/−*^ mice (a model of *MTHFR* polymorphism) exhibited short-term memory deficits, altered expression of synaptic markers and epigenetic enzymes, as well as impaired choline metabolism leading to reduced acetylcholine in the cortex or hippocampus [116]. In addition, folate-deficient diet in these mice leads to altered PS1 mRNA levels, neurotrophic factors, and epigenetic enzymes, as well as increased methionine and reduced SAM/SAH ratios [116]. Similarly, *Mthfr*^*677C> T*^ mice exhibit cerebrovascular deficits such reduced tissue perfusion, decreased vascular density due to increased endothelial cell death, loss of pericytes, and elevated astrocyte and microglia activity [117]. Collectively, these studies indicate that *Mthfr* deficiency may compromise both neuronal and vascular integrity, with many molecular alterations overlapping with known pathogenic mechanisms in AD [118]. FOCM disruption can also affect Aβ and tau phosphorylation, promoting aggregation of amyloid plaques and neurofibrillary tangles as observed in AD brains [119–121]. Studies have shown that low folate, HHcy, and Mthfr deficiency can disrupt Leucine Carboxyl Methyltransferase 1 (LCMT1)-dependent methylation of protein phosphatase 2 A (PP2A), which is a major Ser/Thr phosphatase essential for neuronal homeostasis [120, 121]. LCMT1 promotes the formation of PP2A/Bα heterotrimers by methylating the catalytic subunit of PP2A at Leu-309. These PP2A/Bα heterotrimers, in turn, dephosphorylate tau. Sontag et al. reported that mild Mthfr deficiency in aged *Mthfr*^*+/−*^ mice and severe deficiency in young *Mthfr*^*−/−*^ mice cause region-specific reductions in PP2A and LCMT1, particularly in the hippocampus and cerebellum [122]. Folate deficiency further decreases LCMT1, methylated PP2A, and PP2A/Bα levels [122]. Similar inhibiting effect on methylated PP2A was observed in mice models of HHcy [121]. The resulting downregulation of PP2A/Bα correlates with increased tau and APP phosphorylation, linking FOCM disruption to PP2A hypomethylation as a mechanism contributing to neuronal dysfunction and AD risk [122].

Similar to GWAS study, the significance of mitochondrial FOCM in AD was also reported in fly model of AD. Yu et al. reported that mitochondrial complex I and OCM were disrupted in a fly model of AD overexpressing Aβ-Arc (a toxic form of Aβ) [123]. Supplementation with Nmdmc, the fly orthologue of *MTHFD2L*, alleviated mitochondrial defects, improved motor performance, reduced neurodegeneration, and enhanced survival [123]. Similar protective effects with folinic acid supplementation were observed in fly and cellular model of AD including differentiated SH-SY5Y neuroblastoma cells treated with Aβ1–42 oligomers and SH-SY5Y cells stably expressing APP with the Swedish mutation (APPswe) [123]. Furthermore, this study using UK Biobank data demonstrated that higher folate intake (reflected by increased FOLR3 expression) was associated with a reduced risk of developing AD. Benefit of 5-MTHF supplementation on AD pathology was also observed in a rat model of AD induced by D-gal and AlCl_3_ exposure. 5-MTHF treatment alleviated memory impairment, decreased Aβ1–42 and phosphorylated tau levels, mitigated cholinergic damage and endothelial dysfunction, restored the number and morphology of pyramidal cells in the hippocampal CA1 region, and modulated oxidative stress as well as excitatory amino acid release in this AD mouse model [124].

Another pathway by which FOCM may influence AD risk is through its impact on methionine metabolism. FOCM is tightly linked to methionine cycle and its disruption can cause methionine accumulation, which in turn can lead to neuroinflammation, impaired hippocampal neurogenesis, and cognitive decline [125]. For example, mice treated with L-methionine at twice the daily dietary intake for seven days exhibited these impairments, highlighting the detrimental effects of excessive methionine [125]. Tapia-Rojas et al. showed that methionine-enriched diets in wild type mice promoted AD-like pathology such as increased tau hyperphosphorylation, Aβ accumulation and oligomerization, oxidative stress, synaptic loss, and memory deficits [126]. In another study, high methionine diets in mice also increased Aβ1–40, Aβ1–42, APP, and beta-secretase1 and decreased Aβ-degrading enzymes, including insulin-degrading enzyme and neprilysin [127]. In contrast, methionine restriction reduced Aβ accumulation and improved mitochondrial function in APP/PS1 mice, with more pronounced cognitive benefits in males [128], suggesting it as a potential therapeutic option in AD.

Epigenetic regulators further link FOCM metabolism to AD pathology. NSun2, a noncoding RNA methyltransferase (which requires methyl group from SAM) was reported to be reduced in post-mortem AD brain tissue. NSun2 deficiency exacerbated phenotype in drosophila model of tau toxicity and promoted tau hyperphosphorylation in mice and human induced pluripotent stem cell-derived neuronal cultures. Interestingly, NSun2 overexpression mitigated pathology in tau toxicity model of human neurons [129].

Overall, defects in FOCM can disrupt methionine cycle (leading to HHcy), disrupt DNA synthesis, repair, methylation, and choline metabolism, leading to oxidative stress, excitotoxicity, synaptic dysfunction, and impaired neuronal and vascular integrity. These alterations promote tau hyperphosphorylation, APP/Aβ accumulation, and neuroinflammation, thereby driving AD pathology.

## FOCM and Parkinson’s disease

PD is the second most common neurodegenerative disorder, impacting about 1% of the population above 60 years [130–132]. It is more common and presents at an earlier age in males compared to females [133]. The cardinal symptoms of PD are motor deficits (due to nigrostriatal degeneration) such as bradykinesia, resting tremor, muscle rigidity and postural instability [134, 135]. In addition, frontostriatal-mediated executive dysfunction, including autonomic dysfunction, cognitive/neurobehavioral abnormalities, sleep disorders and sensory abnormalities are also present in many PD cases [134, 135]. The key pathological features of PD are dopaminergic neuronal loss in the substantia nigra pars compacta and depletion of the neurotransmitter dopamine in the striatum [136, 137]. The substantia nigra pars compacta neuronal loss is accompanied by intra-neuronal protein inclusions known as Lewy bodies, which contain several misfolded amyloid proteins, including α-synuclein [138]. In fact, 80% nigrostriatal denervation leads to dopamine deficiency, resulting in motor impairment [137]. While the exact cause of PD still remains unknown, studies have suggested aging, genetic and environmental risk factors may contribute to the risk of PD [130, 139]. Aging is the leading risk factor for PD, with incidence rising sharply in one’s 60’s and then exponentially in subsequent decades of life [139]. Several studies have identified multiple genetic risk factors for PD such as *SNCA* (encoding α-synuclein), parkin RBR E3 ubiquitin protein ligase (*PRKN)*, PTEN-induced kinase 1 *(PINK1)*, *DJ-1* and Leucine-rich repeat kinase 2 (*LRRK2*) [140]. The known genetic risk factors account for approximately 30% of the familial and 3–5% of the sporadic PD cases with LRRK2 being most notable for resulting in sporadic PD [140]. Additionally, environmental risk factors such as pesticides and chemical exposure, traumatic brain injury, virus infection (such as Coxsackie, Japanese encephalitis B, West Nile, and Human immunodeficiency virus), and nutritional deficiencies also contribute to PD risk [141–147]. Emerging evidence suggests an important role of folate metabolism in the pathogenesis of PD with clinical and epidemiological studies increasingly linking FOCM to disease onset and progression [7, 148, 149].

### Clinical and Epidemiological Research

Several studies have demonstrated that PD patients have reduced plasma folate and vitamin B12 levels as well as elevated Hcy compared to healthy individuals [7, 150]. HHcy in PD patients is associated with depression, cognitive deficits, and severity of disease [151–156]. A sex-specific effect of HHcy is also reported, with elevated Hcy associated with greater motor impairment in male PD patients and poorer cognitive performance in female PD patients [157]. Furthermore, elevated Hcy levels can increase the risk of cerebrovascular diseases by causing microvascular damage and endothelial dysfunction, leading to vascular parkinsonism [153, 158].

Genetic polymorphisms in FOCM can contribute to elevated Hcy levels and have been examined for their potential role in PD. Among them, *MTHFR* polymorphisms have been most extensively studied; however, the results remain inconclusive. Diao et al. in a systematic meta-analysis of 21 studies found no association of two common *MTHFR* polymorphisms: C677T and A1298C with PD, however, they reported an association of the *MTHFR* A2756G polymorphism with PD [159]. Another meta-analysis further confirmed the lack of overall association of *MTHFR* C667T polymorphism with PD; however, subgroup analysis by ethnicity revealed a significant association with PD risk among individuals of European ancestry [160]. In addition to *MTHFR* polymorphism, studies examining effect of polymorphisms in other FOCM genes and risk of PD are very limited. A polymorphism in methionine synthase reductase (*MTRR* A1049G) was associated with increased risk of PD [161]. Another study reported that the *MTRR* A66G polymorphism both independently and in combination with the *MTHFR* C677T polymorphism, was associated with a higher risk of PD, while the cytosolic *SHMT* 1420 C > T variant appeared to confer a protective effect [162].

FOCM not only influences PD risk but also contributes to treatment response. Levodopa, a dopamine precursor, is the effective treatment for alleviating motor symptoms of PD [163]. However, several studies have reported that levodopa treatment is associated with elevated plasma Hcy levels, raising concerns about potential Hcy toxicity [151, 164]. Levodopa is metabolized by catechol-O-methyl transferase (COMT) into 3-O-methyldopa generating Hcy. The remethylation of Hcy to methionine requires folate and vitamin B12 as cofactors. Additionally, in the transsulfuration pathway, CBS converts Hcy to cystathionine using vitamin B6 as a cofactor [165]. In PD, the increased metabolic demand imposed by levodopa administration may deplete one or more of these vitamins, thereby contributing to HHcy [165]. Supplementation with folate and vitamin B12 combined with entacapone (a COMT inhibitor) has been shown to reduce plasma Hcy levels in a randomized controlled study, supporting their use as adjunctive therapy to mitigate Hcy-associated toxicity in levodopa-treated patients [166].

There are limited clinical trials targeting FOCM in PD. In a randomized trial (NCT01238926), PD patients were assigned to one of four groups: vitamin supplementation (5 mg/day folic acid, 2,000 mcg/day vitamin B12, and 5 mg/day vitamin B6), exercise intervention, a combination of vitamin supplementation and exercise, or no intervention. The study reported that six weeks of B vitamin supplementation lowered Hcy, exercise improved glutathione, strength, and aerobic capacity, but combining both had no additional benefits [167].

### Experimental Evidence

Experimental models further reinforce the link between Hcy, FOCM, and dopaminergic vulnerability. Paraquat, a widely used herbicide, induces PD-like pathology including dopaminergic neuronal loss [168–170]. A study showed that Mthfr deficiency in mice exacerbated the PD-like symptoms in *Mthfr* ^*+/−*^ mice such as motor function impairments, elevated microglial activity in the substantia nigra, and increased oxidative stress in dorsal striatum suggesting that Mthfr deficiency heightens the vulnerability of substantia nigra and dorsal striatum to paraquat-induced damage [171].

Similarly, HHcy induced by either a folate-deficient diet or by directly infusing Hcy into the mouse brain worsens the neurotoxin 1-methyl-4-phenyl-1,2,3,6-tetrahydropyridine (MPTP) mediated PD symptoms such as dopamine depletion, substantia nigra pars compacta neuronal degeneration and motor dysfunction [82]. Hcy itself has been shown to induce PD-like pathology. In a study by Lee et al., intracerebroventricular administration of Hcy into rat brains for five consecutive days significantly reduced locomotor activity and decreased striatal dopamine levels along with its metabolites, DOPAC and HVA [172]. Similarly, chronic exposure of Hcy for 36 days also elevated striatal Hcy concentrations in mouse brains and decreases dopamine turnover, reduced tyrosine hydroxylase immunoreactivity in the substantia nigra, accompanied by marked reductions in locomotor performance [172]. Another study reported motor deficits associated with significant striatal dopamine depletion and mild oxidative stress following 60 days of chronic Hcy exposure; however, no detectable changes in tyrosine hydroxylase immunoreactivity were observed [173]. In vitro, Hcy can worsen oxidative stress, mitochondrial dysfunction and apoptosis in human dopaminergic cells exposed to the pesticide rotenone or to the pro-oxidant Fe(2+) [82]. Together, these findings indicate that HHcy is toxic to the dopaminergic system and mitigating it could be a potential therapeutic target. In line with this, Jia et al. showed that folic acid supplementation protects against MPTP-induced neurotoxicity in mice by lowering Hcy levels, rescuing dopaminergic neurons, improving motor performance, and reducing oxidative damage in the substantia nigra [174]. Mechanistically, folic acid attenuates activation of the NOD-like receptor thermal protein domain-associated protein 3 (NLRP3) inflammasome, thereby suppressing glial activation and neuroinflammation as well as preserving mitochondrial integrity through p53-peroxisome proliferator-activated receptor γ coactivator 1α (PGC-1α) pathway [174]. The beneficial effect of folic acid supplementation was also observed in drosophila model of PD. Using Parkin-null (Park^*−/−*^*)* flies, a study showed that folic acid supplementation improved the motor dysfunction and decreases hydrogen peroxide and glutathione redox imbalance [175] where as in fly homozygous for Parkin mutation (c00062), folic acid supplementation improved the mortality, locomotory defect, oxidative stress by alleviating mitochondrial dysfunction associated with Parkin mutation [176].

## Other Neurodegenerative Diseases

### Amyotrophic Lateral Sclerosis (ALS)

ALS is the third most common neurodegenerative disease after AD and PD [177, 178]. It is characterized by progressive degeneration of motor neurons in the brain, spinal cord and brainstem, leading to muscle atrophy, weakness, paralysis, and ultimately death, most often due to respiratory failure [177, 178]. The key pathological features of ALS include the loss of pyramidal Betz cells in the motor cortex, anterior horn cells in the spinal cord, and lower cranial motor neurons [179]. About 5–10% of ALS cases are familial [180], which is linked to mutations in more than 30 genes, including Superoxide dismutase 1 (SOD1), Chromosome 9 Open Reading Frame 72 (C9orf72), and TAR DNA-binding protein (TARDBP) [181]. Other risk factors include lifestyle factors such as smoking, low body mass index, and repeated head injuries, as well as environmental exposures like pesticides and the neurotoxin β-methylamino-L-alanine. Additionally, several viral infections have also been associated with increased risk of ALS, such as enteroviruses, human herpesvirus 6, human immunodeficiency virus, and human T-cell lymphotropic virus type 1 [182]. Emerging evidence also suggests that nutrients, including folate, play a role in the development and progression of ALS [183]. Consistent to this, a retrospective study of 69 ALS patients reported insufficient intake of several vitamins, including folate in ALS patients, which was associated with lower Revised Amyotrophic Lateral Sclerosis Functional Rating Scale (ALSFRS-R) functional scores [184]. However, a recent meta-analysis including 812 ALS patients, 2632 controls from 9 studies identified no significant difference in plasma folic acid, Hcy and vitamin B12 levels between ALS patients and controls but showed elevated Hcy in CSF among ALS patients [185]. In contrast, an earlier study reported increased plasma Hcy levels in ALS patients, with elevated Hcy correlating with a potential marker of disease progression [186]. Moreover, a short-term treatment with high-dose vitamin B12, which lowers Hcy has been shown to improve compound motor action potentials in ALS patients [187, 188]. Although evidence linking low folate levels to ALS remains inconsistent, oral vitamin B12 supplementation can significantly elevate plasma folate and B12 levels while reducing Hcy in ALS patients [189]. Notably, folate and B12 supplementation may slow disease progression in the early stages of ALS but with no noticeable effect on the overall survival of the ALS patients [189]. Genetic variants that can modulate Hcy and folate levels have also been explored for their role in ALS. Among them, *MTHFR* C667T polymorphism can increase risk of disease in ALS patients in Caucasian [190]. No effect of other polymorphisms in MTR (A2756G), and SLC19A1 (A80G) was observed in ALS patients [191].

Limited experimental studies examined the relationship between FOCM and ALS. In a mouse model of ALS (SOD1 G93A), 5-MTHF levels are significantly reduced in the plasma, spinal cord, and cortex at the pre-symptomatic stage, whereas Hcy levels are markedly increased following the onset of motor symptoms in the ALS mice [192]. In addition, treatment with folic acid alone, in combination with vitamin B12, significantly reduced Hcy levels in SOD1 G93A mice, delayed disease onset, reduced motor neuron loss, and prolonged survival. Mechanistically, these treatments reduced glial cell activation, inhibited inducible nitric oxide synthase (iNOS) and TNF-α expression in the spinal cord, and reduced apoptosis [193].

### Huntington’s Disease (HD)

HD is a rare, autosomal-dominant neurodegenerative disorder of the central nervous system that typically begins between ages 30 and 50 [194, 195]. The main symptoms are motor disturbances such as dystonia and dysphagia, psychiatric and behavioral disorders (such as anxiety and depression) and cognitive decline [194, 195]. The disease is caused by the CAG trinucleotide expansion in the Huntingtin gene (HTT), which results in an expanded polyglutamine repeat in the Htt protein [196]. The length of the CAG repeats correlates with the survival and age of HD onset, with longer CAG repeats associated with shorter survival and earlier HD onset [197, 198]. The mutated Htt protein forms protein aggregates leading to neuronal damage and disrupt many critical cellular functions in the striatum and cerebral cortex [199]. Although HD is a genetic disease, environmental and lifestyle factors can also affect onset, progression, and severity of disease [200, 201]. The role of FOCM in HD was recognized when a study reported interaction of Htt protein with CBS suggesting the possibility of excitotoxic neuronal damage mediated by HHcy due to CBS deficiency [202]. Later, a small-sized study observed moderate HHcy due to folate deficiency in HD patients compared to controls [203]. In addition, plasma Hcy was elevated in HD patients undergoing long-term treatment compared to untreated HD patients and controls, which was associated with severity of disease in the treated group [204]. Interestingly, an experimental study showed that an extended polyglutamine residue in the tobacco plant leads to the accumulation of mutated Htt protein, affecting plant growth, with several OCM proteins, including GTP cyclohydrolase I (GTPCH, involved in BH4 metabolism in animals) were severely reduced in affected roots. This study further validated their finding in a mouse model of HD (R6/2) which showed reduced levels of GTPCH and DHFR and impaired FOCM and BH4 metabolism in HD mice [205].The significance of FOCM in HD can also be understood by the significant overlap of pathways regulated by vitamin B6, B12 and folate with those obtained from transcriptomic and metabolomic data of HD patients and model systems [206]. Furthermore, treatment with vitamin B6, B12 or folate either alone or in combination interfered with the aggregate formation in yeast model of HD further reinforcing the link between FOCM and HD [206].

### Multiple Sclerosis (MS)

Multiple sclerosis (MS) is an autoimmune neurodegenerative disorder of the central nervous system marked by inflammation, progressive demyelination and neuroaxonal loss [207, 208]. The clinical symptoms include vision loss, numbness or weakness of the limbs, ataxia, bladder or bowel dysfunction and cognitive impairment. MS has four distinct courses: relapsing remitting, primary progressive, secondary progressive, and progressive relapsing [209]. The etiology of MS is multifactorial with several lifestyle and environmental risk factors such as smoking, low sun exposure, vitamin D deficiency and viruses such as Epstein Barr virus, human herpesvirus 6 [210, 211]. Vitamins and other nutritional deficiencies may be involved in the pathogenesis of MS, with growing evidence suggesting the role of FOCM dysregulation in MS [212]. Similar to other neurodegenerative diseases, Hcy levels are elevated in MS patients compared to controls [213–215], which is associated with MS onset in children [216] and cognitive impairments in MS patients [217]. However, data comparing folate and vitamin B12 plasma levels in MS patients and controls are conflicting [213–215, 218, 219]. In contrast to clinical studies that associate HHcy to MS, Peng et al. showed that genetic variants causing HHcy in a large GWAS study are associated with a reduced risk of MS [220], highlighting the need of further investigation into the role of HHcy in MS. In another GWAS study involving 4888 cases and 10,395 controls in the German population, SHMT1 was identified as a novel susceptibility locus for MS, which was further replicated in an independent MS cohort in the Sardinian population [221]. As mentioned in "Folate and its Dietary Sources" Section of this review, SHMT1 converts THF to 5,10-methyleneTHF, which is subsequently required for methionine cycle and maintenance of epigenetic balance. Among common FOCM gene polymorphisms, a meta-analysis revealed a modest association between the *MTHFR* C677T polymorphism and MS, whereas the *MTHFR* A1298C polymorphism showed a significantly stronger association [222]. Genetic association studies in other FOCM genes in MS are limited, with individual studies failing to identify any association of *MTR* A2756G and *MTRR* A66G with MS [222, 223] but showed association of insertion allele of CBS c.844_855ins68bp and the G-allele of RFC1 c.80G > A with an early onset of MS [224]. Clinical trials of folic acid supplementation in MS patients are also limited and showed improvement in Hcy levels, anemia, and quality of life of patients with relapsing-remitting MS with folic acid and vitamin B12 supplementation in one study [225] whereas no effect of folic acid supplementation was observed on disability of MS patients in another study [226]. However, a study using UK biobank data showed that genetically determined folic acid supplement was causally linked to a decrease in MS severity [227]. In experimental autoimmune encephalomyelitis model of MS, AMPKα signaling is required for oligodendrocyte function [228]. As the folate promotes the oligodendrocyte survival and myelination through DHFR-AMPKα axis [51], therefore, folate deficiency, by impairing this DHFR-AMPKα axis, can promote MS development or progression by negatively affecting oligodendrocyte survival and myelin production. Interestingly, folate receptor-β is moderately expressed in chronic lesions of MS patients particularly in areas rich in macrophages [229]. In rat focal model of MS, targeting folate cycle using folate-aminopterin construct (EC2319) reduced folate receptor-β expression, lesion size and attenuated inflammation. Aminopterin is a synthetic folic acid antagonist which can inhibit DHFR. In chronic experimental autoimmune encephalomyelitis, EC2319 mediates its beneficial effect by suppressing inflammatory macrophages. It is important to note that macrophages in animals with experimental autoimmune encephalomyelitis, a model of MS, express folate receptor-β [229, 230] indicating the role of FOCM in pathogenesis of MS.

## Conclusions

**Fig. 2:**
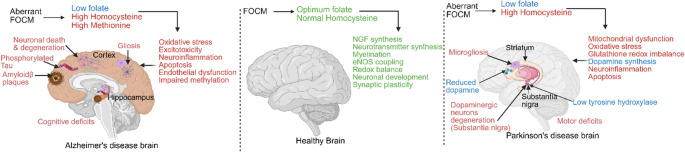
Summary of the role of folate-mediated one carbon-metabolism (FOCM) in healthy, Alzheimer’s disease (AD) and Parkinson’s disease (PD) brains. In the healthy brain, FOCM supports the synthesis of neurotransmitters and nerve growth factors, myelination, eNOS coupling, antioxidant defense, neuronal development, and synaptic plasticity. In AD, impaired FOCM promotes oxidative stress, excitotoxicity, neuroinflammation, endothelial dysfunction, and apoptosis, contributing to neuronal loss, gliosis, tau phosphorylation, amyloid-β accumulation, and cognitive decline. In PD, FOCM disruption causes oxidative stress, mitochondrial dysfunction, glutathione depletion, reduced dopamine synthesis, neuroinflammation, and apoptosis, resulting in increased microgliosis, dopaminergic neuron loss and motor deficits. The figure was created using BioRender

Folate-mediated one-carbon metabolism (FOCM) is essential for brain health throughout the lifespan, extending from its well-known role in neural tube closure and neonatal brain development to its emerging role in cognition, maintenance of synaptic plasticity, neurovascular and neuronal integrity in adults. Disruption of FOCM, whether from genetic variation, dietary folate deficiency, hyperhomocysteinemia, or excess methionine, can lead to oxidative stress, impaired DNA methylation, neurotransmitter imbalance, glial activation, and impaired neuronal function; many of these processes are implicated in the pathogenesis of AD, PD and other neurodegenerative diseases (Fig. 2).

In AD, disruption in FOCM accelerates amyloid-β deposition, tau phosphorylation, cognitive decline, and vascular compromise [113, 114, 116, 117] which can be mitigated by folic acid supplementation in experimental studies [115], and is consistent with clinical evidence showing low folate and elevated homocysteine in patients [6, 8], though supplementation trials remain inconclusive [104, 110]. In PD, folate deficiency and hyperhomocysteinemia worsen motor deficits and nigral dopaminergic loss, with mechanistic links to glutathione depletion, mitochondrial dysfunction, oxidative stress and NLRP3-mediated inflammation [82, 171]. Notably, folic acid supplementation has been shown to ameliorate some of these effects in experimental models [174–176]. While clinical data on folate/folic acid supplementation in PD patients are limited, a combination of folate and vitamin B12 supplementation is increasingly recognized as a strategy to mitigate HHcy-related risks in levodopa-treated patients [166]. In other neurodegenerative such as ALS, MS and HD elevated Hcy levels are commonly reported in patients and experimental models [186, 192, 204, 213]. In ALS, HHcy and folate deficiency are associated with disease progression and increased neuroinflammation, and loss of motor neurons [192, 193]. In MS, aberrant FOCM is linked with disease onset, cognitive impairments, demyelination, and neurodegeneration [51, 216]. Folate appears to play a dual role in MS, while it is required for myelination and neuronal survival, selective inhibition of folate metabolism in macrophages may attenuate inflammation [229]. Although research linking FOCM to HD is limited, aberrant folate metabolism seems to aggravate disease phenotype in HD [205, 206]. There is a need for mechanistic and translational studies to identify the effect of FOCM disruption on distinct brain cell types in several neurodegenerative conditions. It is important to note that excess folate in the context of insufficient vitamin B12 may have a deleterious effect, potentially exacerbating neuropathy and neurodegenerative processes by disrupting methylation balance or masking B12 deficiency [231, 232]. In addition, a limitation of folic acid supplementation studies is that folic acid is a synthetic form of folate that must be converted to active forms in humans. This conversion can be limited and variable, potentially leading to circulating unmetabolized folic acid at higher intake levels. Such variability in metabolism may contribute to inconsistent findings across clinical studies, highlighting the need to measure circulating unmetabolized folic acid in these trials. Therefore, there is also a need for well-designed disease-specific clinical trials that take into consideration the dietary and genetic determinants of FOCM to clarify the contribution of FOCM to neurodegeneration and to help in the development of targeted therapeutic strategies.

In conclusion, folate metabolism is emerging as a critical determinant of brain health and a central link between metabolic imbalance and neurodegeneration. Both deficiency and excess of folate can have adverse effects on brain health, emphasizing the importance of maintaining an optimal balance. Thus, maintaining optimum folate levels may offer opportunities for therapeutic intervention and for reducing vulnerability to neurodegenerative diseases.

## Key References

- Lionaki E, Ploumi C,  Tavernarakis N. One-Carbon Metabolism: Pulling the Strings behind Aging and Neurodegeneration. Cells 2022;11.
  - ○This article is important because it provides a comprehensive and detailed overview of one-carbon metabolism . It also examines the contribution of dysregulated one-carbon metabolism to aging and neurodegeneration.
- Sobral AF, Cunha A, Silva V, Gil-Martins E, Silva R,  Barbosa DJ. Unveiling the Therapeutic Potential of Folate-Dependent One-Carbon Metabolism in Cancer and Neurodegeneration. Int J Mol Sci. 2024;25.
  - ○This important article comprehensively addresses the interaction between folate-dependent one-carbon metabolism and neurodegeneration, detailing multiple underlying mechanisms. It also outlines therapeutic approaches and considerations for targeting this pathway in future studies on neurodegenerative diseases.
- Reagan AM, Christensen KE, Graham LC, Bedwell AA, Eldridge K, Speedy R, Figueiredo LL, Persohn SC, Bottiglieri T, Nho K, et al. The 677C > T variant in methylenetetrahydrofolate reductase causes morphological and functional cerebrovascular deficits in mice. J Cereb Blood Flow Metab*. *2022;42:2333-50.
  - ○This article is important because it provides direct experimental evidence that the common human MTHFR 677C>T variant causes structural and functional cerebrovascular deficits in vivo, using a knock-in mouse model that recapitulates the human enzyme defect.
- Yu Y, Chen CZ, Celardo I, Tan BWZ, Hurcomb JD, Leal NS, Popovic R, Loh SHY, Martins LM. Enhancing mitochondrial one-carbon metabolism is neuroprotective in Alzheimer's disease models. Cell Death Dis. 2024;15:856.
  - ○An important contribution demonstrating that increasing one-carbon metabolism is a promising therapeutic strategy for delaying disease progression and reducing disease severity in experimental models of Alzheimer's disease. It also shows that higher folate intake in humans is associated with a reduced risk of AD and its pathology.
- Houlihan KL, Keoseyan PP, Juba AN, Margaryan T, Voss ME, Babaoghli AM, Norris JM, Adrian GJ, Tovmasyan A,  Buhlman LM. Folic Acid Improves Parkin-Null Drosophila Phenotypes and Transiently Reduces Vulnerable Dopaminergic Neuron Mitochondrial Hydrogen Peroxide Levels and Glutathione Redox Equilibrium. Antioxidants (Basel)2022;11.
  - ○This important study demonstrates the therapeutic effect of folic acid supplementation in an vivo model of Parkinson’s disease caused by loss-of-function parkin mutations, linking folate-mediated one-carbon metabolism to parkin-associated neurodegeneration.

## Data Availability

No datasets were generated or analysed during the current study.

## Abbreviations

5-MTHF: 5-Methyltetrahydrofolate
Aβ: amyloid-beta
ALSFRS-R: Revised Amyotrophic Lateral Sclerosis Functional Rating Scale
AD: Alzheimer’s disease
ALDH1L2: 10-formyl-THF dehydrogenase
ALS: Amyotrophic lateral sclerosis
AMPK: Adenosine monophosphate-activated protein kinase
ApoE: Apolipoprotein E
ASD: Autism spectrum disorder
APP: Amyloid precursor protein
ATP: adenosine triphosphate
BDNF: Brain-derived neurotrophic factor
BH2: Dihydrobiopterin
BH4: Tetrahydrobiopterin
CBS: Cystathionine beta-synthase
COMT: Catechol-O-methyl transferase
C9orf72: Chromosome 9 open reading frame 72
CSF: Cerebrospinal fluid
DHF: Dihydrofolate
DHFR: Dihydrofolate reductase
dTMP: Deoxythymidine monophosphate
dUMP: Deoxyuridine monophosphate
eNOS: Endothelial nitric oxide synthase
ERK: Extracellular signal-regulated kinase
FR: Folate receptor
FRα: Folate receptor alpha
FOCM: Folate-mediated one-carbon metabolism
GTPCH: GTP cyclohydrolase I
GPx: Glutathione peroxidase
GWAS: Genome-wide association study
HHcy: Hyperhomocysteinemia
Hcy: Homocysteine
HD: Huntington’s disease
HTT: Huntingtin
IL-6: Interleukin-6
iNOS: Inducible nitric oxide synthase
LCMT1: Leucine carboxyl methyltransferase
LRKK2: Leucine-rich repeat kinase 2
MAPK: Mitogen-activated protein kinase
MAT2A: Methionine adenosyltransferase 2 A
MTHFD1: Methylenetetrahydrofolate dehydrogenase 1
MTHFD2: Methylenetetrahydrofolate dehydrogenase 2
MTHFD1L: Methylenetetrahydrofolate dehydrogenase 1-like
MTHFR: Methylenetetrahydrofolate reductase
MTR: Methionine synthase
MS: Multiple sclerosis
MTRR: Methionine synthase reductase
NAD: Nicotinamide adenine dinucleotide
NGF: Nerve growth factor
NF-κB: Nuclear factor kappa-light-chain-enhancer of activated B cells
NMDA: N-methyl-D-aspartate
NO: nitric oxide
NTD: Neural tube defect
OCM: one-carbon metabolism
PARP: poly-ADP-ribose polymerase
PD: Parkinson’s disease
PCFT: Proton-coupled folate transporter
PINK1: PTEN-induced kinase 1
PRKN: Parkin RBR E3 ubiquitin protein ligase
PS1: Presenilin 1
ROS: Reactive oxygen species
RFC: Reduced folate carrier
SAH: S-adenosylhomocysteine
SAM: S-adenosylmethionine
SHMT1: Serine hydroxymethyltransferase 1
SHMT2α: Serine hydroxymethyltransferase 2 alpha
SOD1: Superoxide dismutase
TARDBP: TAR DNA-binding protein
THF: Tetrahydrofolate
TNF-α: Tumor necrosis factor alpha
TYMS: Thymidylate synthase
